# Sterigmatocystin in foodstuffs and feed: aspects to consider

**DOI:** 10.1080/21501203.2018.1492980

**Published:** 2018-07-09

**Authors:** Carla Viegas, Janne Nurme, Elena Piecková, Susana Viegas

**Affiliations:** a H&TRC- Health & Technology Research Center, ESTeSL- Escola Superior de Tecnologia da Saúde, Instituto Politécnico de Lisboa, Lisbon, Portugal; b Centro de Investigação em Saúde Pública, Escola Nacional de Saúde Pública, Universidade NOVA de Lisboa, Lisbon, Portugal; c Faculty of Medicine, Slovak Medical University, Bratislava, Slovakia

**Keywords:** Sterigmatocystin, foodstuffs and feed, climate change, increased risk, public health

## Abstract

Sterigmatocystin (STC) is a possible human carcinogen (2B) according to International Agency for Research on Cancer classification and has been associated with immunotoxic and immunomodulatory activity, together with mutagenic effects. It might be found in numerous substrates, from foods and feeds to chronically damp building materials and indoor dust. Although European Food Safety Authority concluded that the exposure to STC to be of low concern for public health, reinforces the need of data concerning exposure of European citizens. Climate change can represent an increased risk of exposure to STC since it is a crucial factor for agro-ecosystem powering fungal colonisation and mycotoxin production This aspect can represent an increased risk for European countries with temperate climates and it was already reported by the scientific community.

## 1. Background

Food and feed can be contaminated with a mixture of mycotoxins, toxic secondary metabolites produced by fungal species. Fungal invasion and consequent mycotoxin production occurs both in the field and/or during the storage of crops (de Nijs et al. 2016). The level of contamination is dependent on many factors such as fungal interactions, type of crops and environmental factors (Battilani et al. 2012; Van der Fels-Klerx 2016). In food, other aspects can influence the profile and levels of the mycotoxins present, namely, industrial processes involved and, after being acquired, household preparation process (Nijs et al. 2016). In addition, we should consider that mycotoxins are extremely difficult to eliminate from food even after the cooking process because they are quite stable molecules. All this explains why mycotoxins can be present in the food or environment long after death and disintegration of the toxic fungus (Peraica et al. 1999; Halstensen 2008; Alborch et al. 2011; Viegas et al. 2015).

## 2. *Aspergillus* section *Versicolores*

The identification of aspergilli down to the species level was traditionally based on the morphological features (Raper and Fennel 1965). More recently, Houbraken et al. (2014) and Hubka et al. (2014) proposed the system comprising four subgenera (*Aspergillus, Circumdati, Fumigati, Nidulantes*) with 20 sections based on phylogenetic approach. The official fungal DNA barcode applying ITS locus (Schoch et al. 2012) has seemed to be insufficient for accurate identification of aspergilli and their sexual morphs, so, additional marker analyses were developed: calmodulin gene(*CaM*), beta-tubuline (*BenA*) and RNA polymerase II second largest subunit (*RPB2*) sequencing, along with extrolite spectrum data as well (Samson et al. 2014; Frisvad 2015). Nowadays, 17 species are assigned to the *Aspergillus* section *Versicolores:: A. amoenus, A. austroafricanus, A. creber, A. cvjetkovicii, A. fructus, A. griseoaurantiacus, A. hongkongensis, A. jensenii, A. pepii, A. protuberus, A. puulaauensis, A. subversicolor, A. sydowii, A. tabacinus, A. tennesseensis, A. venenatus* and *A. versicolor* (Jakšić Despot et al. 2017). According to the works by Chen et al. (2016) and Hubka et al. (2016), the section *Versicolores* was merged to the section *Nidulantes* as the *A. versicolor* clade, to maintain monophyly of *Aspergillus*. The newest combined phylogeny of aspergilli at the section *Versicolores* based on GenBank sequences was published by Jakšić Despot et al. (2017).

Along to the typical aspergillum – a biseriate head on a long stipe, several *A. versicolor* and *A. sydowii* isolates produce diminutive conidial heads resembling penicillate conidiophores. The recommended sexual name *Emericella* should be used for species in the section *Versicolores. Aspergillus versicolor* (Vuil.) Tirab. was historically the most commonly reported representative of the entire section. Its herbarium strain CBS 538.65 bears ITS barcode EF652442, *BenA* = EF652266, *CaM *= EF652354, *RPB2* = EF652178, followed by *A. sydowii* (Bainier and Sartory) Thom and Church, herb.: IMI 211,384, ITS barcode: EF652450, *BenA* = EF652302, *CaM *= EF652390, *RPB2* = EF652214 (Samson et al. 2014). So, that is why *A. versicolor* strains were reported growing worldwide, prospering in many habitats, including foodstuffs and feedstuffs as well as indoor environments, and being implicated in various human and animal health hazards, from mycoses to mycotoxicoses due to production of sterigmatocystin (STC). Of note, it has been already reported that their high prevalence in some specific occupational environments is a consequence of this evidence, such as swineries (Sabino et al. 2012; Viegas et al. 2013).

An extensive search was performed to identify scientific papers, available in different scientific databases and published after 2010, reporting *Aspergillus* section *Versicolores* presence in foodstuffs and feed samples. These species have been reported in several foodstuffs such as cocoa (Egbuta et al. 2014), maize (Makun et al. 2010), date fruits (Al-Bulushi et al. 2017), dry-cured meat products (Sonjak et al. 2011), coffee beans (Batista et al. 2003; Viegas et al. 2017), wheat (Tančinová and Labuda 2009; Al-Hazmi 2010), wheat and wheat products (Riba et al. 2008; Piotrowska 2013), rice (Lima et al. 2000; Aydin et al. 2011), cereals (Tabuc et al. 2010; Toffa et al. 2013), peanut (Toffa et al. 2013), ready-to-use vegetable salads (Kocić-Tanackov et al. 2010), vegetables (Accensi et al. 2004), honey (Kačániová et al. 2012), apples with rotting (Tančinová et al. 2013), puerh tea (Haas et al. 2013), frozen chicken (Darwish et al. 2016), dried raisins (Alghalibi and Shater 2004) and spice Kashmiri chilli mild (Hammami et al. 2014). Several different feeds were reported to be contaminated, namely, maize grain (Chemining’wa et al. 2009; Ayalew 2010), fish feed (Barbosa et al. 2013), feed mixture (Magnoli et al. 2002; Accensi et al. 2004; Labuda and Tančinová 2006), silage (El-Shanawany et al. 2005), soybean (Kačániov 2003), cereals (Accensi et al. 2004; Tabuc et al. 2009, 2011), raw materials like corn and barley rootlets (Rosa et al. 2008), soybean meals (Al-Seeni 2012) and sorghum (Silva et al. 2000) (Table 1).

**Table 1.** *Aspergillus* section *Versicolores* presence in food and feed samples.

Setting	Food or feed sampled	Number of samples	Analytical methods	Results	Reference
Rough rice warehouse from Rio Grande do Sul Brazil	Polished rice	30	Plated onto Potato dextrose agar; plates were incubated for 7 days at 25°C	Prevalence 6.6%	Lima et al. (2000)
	Rice bran	30			
	Rice hulls	30			
Freshly harvested and stored sorghum from the State of São Paulo, Brazil	Sorghum	140	Inoculated onto potato dextrose agar; Incubated at 25°C for 5 days and observed daily	1.4% prevalence	Silva et al. (2000)
Mixed feed production plant from Cordoba, Argentina	Poultry feed	120	Inoculated onto DRBC agar; incubated in darkness at 28 for 7 days	1.0 × 10^3^–5.0 × 10^3^ (CFU g^−1^)	Magnoli et al. (2002)
Eleven municipalities supermarkets from Brazil	Processed coffee beans (*Coffea arabica* L.)	45	Plated directly onto filter paper moistened with sterile distilled water; 15 were plated without prior hypochlorite disinfection and 15 were disinfected with 1% hypochlorite for 2 min	3 isolates	Batista et al. (2003)
Agricultural companies in the years 2002 and 2003 from Slovak Republic	Soybean	25	Inoculated onto Czapek-Dox agar with streptomycin; incubated at 25°C 10 days	2 isolates	Kacaniova (2003)
Local factories	Mixed feeds	147	Inoculated onto malt extract agar; incubated at 28°C for 7 days	Prevalence 9.5%	Accensi et al. (2004)
	Cereals	117		Prevalence 14.5%	
	Vegetables	36		Prevalence 8.3%	
Dried fruits from shops and marketsin Yemen Republic	Dried raisins, figs and dates	20	Inoculated to 1% glucose and 40% sucrose-Czapek’s agar media; Incubated at 28°C for 10 days	1 isolation from dried raisins	Alghalibi and Shater (2004)
Farms, mills and retail markets at Assuit and Sohag governorates from Egypt	Silage	40	Inoculated onto abouraud’s dextrose agar with chloramphenicol and cyclohexemide; -incubated at 28°C for 1–2 weeks	3 isolates	El-Shanawany et al. (2005)
Poultry farms from Slovakia	Feed mixture	108	Inoculated onto Dichloran chloramphenicol peptone agar (DCPA); incubated at 25°C for 5–7 days; conidial suspensions inoculated onto Czapek Yeast Extract Agar (CYA), Czapek Yeast Extract Agar CY20S and Malt extract agar (MEA), and incubated in the dark at 25°C for 7–14 days	Occurrence in 13 samples with 33 isolates	Labuda and Tančinová (2009)
Setif region	Wheat and wheat products	30	Inoculated onto Dichloran Rose Bengal Chloramphenicol Agar (DRBC) Agar; incubated for 5‒7 days at 28°C in the dark; colonies sub-cultured on malt extract agar; allowed to grow at 28°C for 7‒10 days	Frequency ranged from 10% to 28%	Riba et al. (2008)
Tizi Ouzou region		31			
Flour mill		12			
Semolina mill and samples from Algeria region		12			
Cattle dairy feedfrom Rio de Janeiro State, Brazil	Raw material (corn, brewer’s grain, barley rootlets, cotton flour, pelletised citric pulp)	106	Inoculated onto DRBC; incubated at 28°C for 7 days	Corn prevalence11.8% and barley rootlets prevalence 8.1%	Rosa et al. (2008)
	Finished cow feed	24		-	
Nine agro-ecological zones from Eastern Kenya	Maize grain	5 farmers per ecological zone	Inoculated onto Czapek dox agar; incubated at 25°C for 7–10 days	Positive results in 6 zones, percentage ranging from 0.6% to 6.6%	Chemining’wa et al. (2009)
Cereal samples from Southeastern Romania	Corn	54	Plated onto malt agar and malt salt agar; colonies were counted after 3, 5, and 7 days of culture at 25°C and 31°C	Prevalence of 46.2%	Tabuc et al. (2009)
	Wheat	35		Prevalence of 22.8%	
	Barley	21		Prevalence of 9.4%	
Mills from Slovakia	Wheat bran	56	Inoculated onto Dichloran chloramphenicol peptone agar (DCPA); 5–7 days of incubation at 25°C	Occurrence in 5 samples with 8 isolates	Tančinová and Labuda (2009)
Wheat from different production areas and sold Jeddah market, Saudi Arabia	Wheat grain	30	Inoculated onto potato dextrose agar (PDA) ; incubated at 25°C in dark for 7 days	2 × 10^2^ colony/g 3 × 10^3^ colony/g	Al-Hazmi (2010)
2004/2005 harvest in Ethiopia	Maize grain	17	Inoculated onto Dichloran Glycerol (DG)18 agar; Incubated at room temperature for 1–2 weeks	Surface prevalence 23.5%	Ayalew (2010)
Fresh ready-to-use salads made of different types of vegetables in Serbia	Salads	17	Inoculated to DRBC agar; Incubated at 25°C for 5–7 days	Prevalence of 1.78%	Kocić-Tanackov et al. (2010)
Maize field, market and storage facilities from three States in Nigeria	Five different food commodities	343	Inoculated onto PDA; incubated 5–7 days at 28°C	2 isolates	Makun et al. (2010)
Cereal samples from different locations in Banat region, Romania	Cereal grain (corn, wheat, barley, oats)	56	Applied onto malt and agar with 6% NaCl.; incubated for 5–7 days at 25°C	10.7% prevalence	Tabuc et al. (2010)
Two rice-growing areas Uzunkopru and Ipsala in the Thrace region ´from Turkey	Rice	100	Inoculated onto DRBC agar; incubated at 25°C for 5–7 days; Colonies sub-cultured on malt extract agar incubated at 25°C for 5–7 days	2 positive samples	Aydin et al. (2011)
Slovenian meat-processing plant	Dry-cured meat products	75 items of different dry-cured meat products	Inoculated to MEA and to MEA with 5% NaCl; isolates inoculated onto MEA and grown for 7 days at 25°C in the dark	2 isolates	Sonjak et al. (2011)
Cereals produced in the South East part of Romania between 2008 and 2010	Feed cereals (maize, wheat, barley, oats, rye, soya, sunflower, colza, rice, triticale)	86	Plated onto malt agar mediumand malt salt agar medium; incubated 7 days at 25°C and 31°C	2008 (*n* = 42)38.0% 2009 (*n* = 32)31.2% 2010 (*n* =12)58.3%	Tabuc et al. (2011)
Soybean meal collected from fodder markets at Jeddah, Saudi Arabia	Soybean meals	20	Inoculated onto Czapek-Dox agar with 1% streptomycin; incubated at 25°C 10 days	1.4 × 10^2^ count/g	Al-Seeni (2012)
Bee-keepers (apiary honey) from Slovakia and other countries	Honey	53	The cultures grew under speciﬁc conditions on Czapek-Dox agar and Malt agar	3 positive samples with frequency 6.82%	Kačániová et al. (2012)
Two tilapia farmsin the Rio de Janeiro State, Brazil	Fish feed	60	Inoculated onto DRBC and DG18; Incubated at 25°C for 5‒7 days	16% relative density	Barbosa et al. (2013)
Different stores and markets in south-western Nigeria	Processed cocoa	22	Inoculated onto PDA, OAES Aohio agricultural experimental station agar, MEA and CYA; incubated 4–7 days at 25°C	2.0 × 10^4^ (Cfu/g) 4.6%	Egbuta et al. (2013)
Tea shops from the province of Yunnan in south-western China	Pu-erh tea	36	Plated onto MEA, Sabouraud Glucose Agar and DG18; Incubated at 25°C 7–14 days	2.8% prevalence	Haas et al. (2013)
Cereal breakfast products from Lodz (Poland) markets	Cereal snacks with cinnamon, cornflakes, cornflakes with nuts and honey, multi-cereal products, cereal products with chocolate and muesli with dried fruit, nuts, cereal and coconut flakes	15	Inoculated onto DRBC agar and DG18; incubated at 25°C for 7 days	Cereal snacks with cinnamon ‒ 37%;cornflakes with nuts and honey ‒ 24%; multi-cereal products ‒ 15%; muesli ‒ 20%; cereal products with chocolate ‒ 50% and 10%	Piotrowska (2013)
Apples with the characters of rotting bought in the commercial network from Slovakia	Rotting apples	30	Roducers of rotting were inserted directly onto MEA Cultivated 5–7 days in the dark at 25 ± 1 °C; *Aspergillus* sp. isolated to CYA, MEA, CY20S, CREA creatine sucrose agar and YES; cultivation proceeded for 5–7 days in the dark at 25 ± 1°C	1 isolate	Tančinová et al. (2013)
Cereals and peanut from local markets from Republic of Niger	Cereals (rice, maize, sorghum, millet) and peanut	81	Plated onto PDA, CYA, MEA; Incubated MEA 30°C, PDA 27°C, CYA 25°C for 7 days	4 strains	Toffa et al. (2013)
Powdered spices from Qatar local markets	Spices: chili, Kashmiri chili hot, Kashmiri chili mild, basil, oregano, ginger, curry, cumin, turmeric, tandoori masala, garam masala, black pepper, garlic and coriander	14	Inoculated onto DRBC agar; Incubated at 28°C 5 days	In Kashmiri chili mild 3 isolates	Hammami et al. (2014)
Frozen chicken from different localities in Zagazig city, Egypt	Breast	20	Culturing on malt extract agar media and Czapeck-Dox agar with 5% Nacl; incubation in dark at 25°C for 5–7 days	10%	Darwish et al. (2016)
	Thigh	20		10%	
	Gizzards	20		14–15%	
	Livers	20		4–5%	
Several date palm from Oman region	Date palm (Tamar stored dates)	3 date fruits from each cultivar.	Illumina MiSeq Sequencing Analysis	Frequency of occurrence in Khenizi mesocarp 0.32 and skin 0.065	Al-Bulushi et al. (2017)
Green coffee beans from *Coffea arabica* (Arabica coffee) and *Coffea canephora* var. *robusta* (Robusta coffee) to be roasted in Portugal	Samples from different countries of origin	28	The washed supernatant (100 μl) was plated in MEA and DG18; incubated at 27°C for 5 to 7 days.	6.1 prevalence	Viegas et al. (2017)

## 3. STC production ability

Mycotoxin STC synthesis is restricted to species in four sections in *Aspergillus* (*Ochraceorosei, Versicolores, Nidulantes* and *Flavi*) (Rank et al. 2011). Most of the aspergillus species from the section *Versicolores* are able to produce STC, namely, *A. amoenus, A. creber, A. cvjetkovicii, A. fructus, A. griseoaurantiacus, A. hongkongensis, A. jensenii, A. pepii, A. protuberus, A. puulaauensis, A. subversicolor, A. tennesseensis, A. venenatus* and *A. versicolor* (Jurjievic et al. 2013; Visagie et al. 2014; Jakšić Despot et al. 2017). According to Frisvad (2015), the metabolic profile is unique for each fungal species entity with a high degree of chemoconsistency among different isolates of the particular species. But, so far, at the section *Versicolores*, there are chemical markers characterised just for *A. versicolor* and *A. sydowii* (Samson et al. 2010). Liquid Chromatography Mass Spectrometry (LC-MS) LC/MS-based methods proved to be accurate to identify chromatograms of fungal extracts in general. At the moment, some other extrolites of *A. versicolor* were found, like polyketides (Lee et al. 2010), stephacidin A and notoamide B (Greshock et al. 2008) or kipukasins (Jiao et al. 2007), respectively.

STC and dihydrosterigmatocystin are the penultimate precursors of aflatoxins – polyketide-derived furanocoumarins. It has been demonstrated that 25 identified genes clustered within a 70-kb DNA region in the chromosome are involved in their biosynthesis (Townsend 1997). The homologous genes and their corresponding enzymes acting in each bioconversion step in the biochemical pathway common to aflatoxins and STC were described later on as well (Yu et al. 2004). For example, in *A. nidulans*, the last in the row crucial gene seems to be *stcP* encoding O-methyltransferase B required for the conversion of dimethylsterigmatocystin to STC (Kelkar et al. 1996).

## 4. STC toxicity

STC is a possible human carcinogen (2B) according to IARC classification (McConnell and Garner 1994) and showed immunotoxic and immunomodulatory activity (Liu et al. 2014), together with mutagenic effects (Gao et al. 2015). It might be found in numerous substrates, from foods and feeds to chronically damp building materials and indoor dust.

In 2013, the European Food Safety Authority (EFSA) was asked by the European Commission to deliver a scientific opinion on STC in food and feed. The Panel on Contaminants in the Food Chain (CONTAM) from EFSA was responsible for this opinion. However, due to the absence of exposure data for the European population, the margin of exposure approach for substances that are genotoxic and carcinogenic could not be applied for STC, and therefore, the risk of STC for human health was not characterised.

Despite this, it was possible to collect all the available information related to STC toxicokinetics, toxicity, mode of action and dose-response assessment by comparing with aflatoxin B1. The following information was available in the EFSA Journal and, more precisely, in the scientific opinion on the risk for public and animal health related to the presence of STC in food and feed (EFSA Panel on Contaminants in the Food Chain (CONTAM) 2013).

This report concludes the exposure to STC to be of low concern for public health based on the relative carcinogenic potency of STC and AFB1 and exposure data. However, the need of data concerning exposure of European citizens was also mentioned (EFSA Panel on Contaminants in the Food Chain (CONTAM) 2013).

### 4.1. STC toxicokinetics

There is limited information available related to STC toxicokinetics. However, the accessible data suggests that absorption of STC is limited following oral exposure.

In the same way, data on the biotransformation of STC is also insufficient. Few studies published to date indicate that phase I metabolism of STC comprises cytochrome P450 (CYP450)-mediated formation of a reactive epoxide as well as monohydroxylation and dihydroxylation reactions. In a more detailed manner, STC is metabolised in the liver and lung by various CYP450 enzymes into different hydroxymetabolites and its reactive exo-epoxide that readily forms DNA adducts (EFSA Panel on Contaminants in the Food Chain (CONTAM) 2013; Walkow et al. 1985).

As phase II metabolites, a glucuronide of STC and of monohydroxy-STC has been observed and reported, together with a sulphate conjugate of monohydroxy-STC and a glutathione adduct of a monooxygenated STC. Excretion of both conjugated parent STC and its hydroxylated metabolites occurs via bile and urine. Nevertheless, the structure of most of these metabolites is not completely known and more research is necessary to allow the availability of more detailed information (Walkow et al. 1985; EFSA Panel on Contaminants in the Food Chain (CONTAM) 2013).

### 4.2. Toxicity of STC

Due to the structural similarities, aflatoxins and STC share relevant toxic effects, including genotoxicity and carcinogenicity (Miller and Trenholm 1994; EFSA Panel on Contaminants in the Food Chain (CONTAM) 2013). However, in contrast to aflatoxins, only limited information on occurrence and toxicity of STC is available.

Liver and kidneys are the target organs of acute toxicity. However, the acute oral toxicity is relatively low (range between 120 and 166 mg/kg body weight). STC is hepatotoxic in rat, mouse, monkey and guinea pig. The incidence of hepatocellular necrosis and haemorrhages increases with dose and duration of exposure. In the kidney, hyaline degeneration, tubular necrosis and haemorrhages were described in rats and/or monkeys exposed to STC (Purchase and Van Der Watt 1969; EFSA Panel on Contaminants in the Food Chain (CONTAM) 2013).

Results from *in vivo* and *in vitro* studies suggest that STC may have also immunomodulatory activity, but strong conclusions cannot be drawn (Huang et al. (2002), Xing et al. (2005), and Zhang et al. (2012) cited in EFSA Panel on Contaminants in the Food Chain (CONTAM) 2013)).

STC is mutagenic in both bacterial and mammalian cell assays after metabolic activation. Subsequently, STC induces chromosomal damage both *in vitro* and *in vivo* in experimental animals (Curry et al. 1984; Ueda et al. 1984; Mori et al. 1986; Crofton-Sleigh et al. 1993; Abdel-Wahhab et al. 2005).

Various studies aimed to compare the genotoxicity of STC and AFB1. However, the uncertainty regarding their actual concentration in the test system, the efficiency of the activation/detoxification metabolic routes and the repair rate of induced lesions does not allow a direct comparison of the relative mutagenic potency of these mycotoxins (EFSA Panel on Contaminants in the Food Chain (CONTAM) 2013).

In previous studies, tumourigenicity of STC was observed after oral, intraperitoneal, subcutaneous and/or dermal administration in the animal species tested (rat, mouse, Mongolian gerbils, monkey and fish). After oral exposure, premalignant and malignant lesions such as hepatocellular carcinomas (HCC), haemangiosarcomas in the liver, angiosarcomas in the brown fat, lung adenomas and incidental findings in other organs were reported (EFSA Panel on Contaminants in the Food Chain (CONTAM) 2013).

Base on the available information, the CONTAM Panel of EFSA concluded that STC is genotoxic and carcinogenic (EFSA Panel on Contaminants in the Food Chain (CONTAM) 2013).

Additionally, a study developed by Miller et al. (2010), which considered exposure of STC via the indoor environment, concluded that following inhalation (intratracheal installation) of STC, a non-specific but severe inflammatory response of the lung tissue was observed. Similarly, severe cytotoxic and inflammatory damage of lung tissue as well as breaking down of self-cleaning mechanism of airways in rats *in vivo* were observed after intratracheal instillation of STC containing complex extrolites of an *A. versicolor* strain of indoor origin in the studies by Piecková et al. (2011, 2015).

### 4.3. STC mode of action

The mode of action of STC can be described as follows. Phase I metabolism results in metabolic activation that promotes the formation of N7-guanyl DNA adducts. These adducts are likely to be responsible for the STE mutagenic effects (Essigmann et al. 1979, 1980). A dose-dependent formation of DNA adducts of STC was found in the concentration range between 1 and 3 mg STC per liver (Reddy et al. 1985; cited in EFSA Panel on Contaminants in the Food Chain (CONTAM) 2013).

STC induces cytotoxicity, inhibition of cell cycle and mitosis, as well as an increased formation of reactive oxygen species (ROS) and lipid peroxidation *in vivo* (Kawai et al. 1984; Ueno et al. 1995; Xie et al. 2000; Sivakumar et al. 2001; Bünger et al. 2004).

The conclusion made by EFSA Panel on Contaminants in the Food Chain (CONTAM) (2013) was that the genotoxicity of STC is based on the formation of DNA adducts that, if unrepaired, increase the likelihood of mutation fixation. Moreover, when comparing with AFB1, most *in vitro* studies with purified DNA indicate that the level of induced N7-guanyl adducts is higher after AFB1 than STC exposure, supporting the view that AFB1 is a more potent liver carcinogen than STC. Various *in vitro* and *in vivo* investigations have demonstrated that STC exerts cytotoxicity, inhibition of cell cycle and mitosis, as well as an increased ROS formation and lipid peroxidation *in vivo*. However, most of the *in vitro* assays have been conducted with rather high STC concentrations, not representing the real human exposure scenario that should be a chronic exposure. Therefore, the observed effects of those studies have to be interpreted with caution not allowing to make conclusions regarding the potential adverse effects of (low dose) dietary exposure to STC (EFSA Panel on Contaminants in the Food Chain (CONTAM) 2013).

More recently, a study developed by Wang et al. (2015) tried to confirm that STC exposure is a risk factor for oesophageal cancer and that STC may induce DNA damage and G2 phase arrest in immortalised human oesophageal epithelial cells (Het-1A). Indeed, the study developed allowed to conclude that STC can induce different cell cycle arrest in primary human oesophageal epithelial cells and immortalised human oesophageal epithelial cells *in vitro* (Wang et al. 2015).

In 2017, Jiang and co-authors aimed to investigate whether checkpoint adaptation occurs in GES-1 Cellosaurus cell line (GES-1) cells treated with STC. The results suggested that STC induces an initial G2 arrest that is subsequently followed by G2 phase checkpoint adaptation, which may potentially promote genomic instability and result in tumorigenesis (Jiang et al. 2017).

Additionally, a study developed by Huang et al. (2014) in human pulmonary cells *in vitro* observed that STC induced DNA damage and affected key proteins involved in cell cycle regulation to trigger genomic instability, which may be a potential mechanism underlying the developmental basis of lung carcinogenesis.

### 4.4. Dose–response modelling

Despite the evidence on genotoxicity and carcinogenicity, only a limited tumourigenicity database was available for dose–response assessment since most of the studies published have several limitations that do not allow to be used for dose–response modelling (EFSA Panel on Contaminants in the Food Chain (CONTAM) 2013).

Being aware of this limitation, the CONTAM Panel of EFSA compared the carcinogenic potency of STC and AFB1 in the BMD10 values. After the comparison of the BMD10 of STC for the occurrence of haemangiosarcomas and that of AFB1 for the occurrence of HCC, the CONTAM Panel concluded the carcinogenic potency of STC is approximately three orders of magnitude lower than that of AFB1 (EFSA Panel on Contaminants in the Food Chain (CONTAM) 2013).

## 5. Food and feed contamination already reported

The natural occurrence of STC in foodstuffs and feed has been reported in a limited number of surveys (Table 2). Reports from year 2000 to 2017 have presented STC contamination in several foodstuffs such as rice (Sawane and Sawane 2014; Mo et al. 2015; Rofiat et al. 2015; Bertuzzi et al. 2017), bread (Veršilovskis and Bartkevičs 2012), wheat bran (Tančinová and Labuda 2009), grain (Veršilovskis et al. 2008a; Mo et al. 2015;), maize (Warth et al. 2012; Mo et al. 2015;), groundnuts (Warth et al. 2012), peanut seed (Youssef et al. 2008), coffee bean (Bokhari and Aly 2009; Culliao and Barcelo 2015), beer (Veršilovskis et al. 2008b), cheese (Veršilovskis et al. 2009), cereal grains, cereal products (Mo et al. 2015) and almond seed (Yassin et al. 2013). Additionally, some reports in animal feed found contamination in grass (Nichea et al. 2015), feed mixture (Labuda and Tančinová 2006; Warth et al. 2012), wheat, corn, barley, soybean, sunflower cake (Kovalenko et al. 2011), silage (El-Shanawany et al. 2005), straw and hay (Mol et al. 2014).

**Table 2.** Sterigmatocystin prevalence in foodstuffs and feed samples.

Setting	Food and feed sampled	Number of samples	Extraction method	Analytical method	Results (quantitative and qualitative)	Reference
Assuit and Sohag farms, mills and retail markets	Silage	40	Chloroform extract	TLC	2 samples	El-Shanawany et al. (2005)
Poultry farms	Feed mixture	108 samples, 5132 isolates	chloroform:methanol (2:1, v/v)	TLC	10 isolates	Labuda and Tančinová (2006)
Different parts of Latvia	Grain samples	1. In 2006 95 samples. 2. In 2007 total 120 samples	84% acetonitrile in water	-LC–MS/MS analysis -An electrospray ionization (ESI)-	1. 13.7% (13 samples) from 2006 (0.7 to 83 µg/kg) 2. 35% (42 samples) from 2007 (1 to 47 µg/kg)	Veršilovskis et al. (2008a)
Latvia local supermarkets	Beer (9 dark, 17 light)	26	Acetonitrile:water (10:90, v/v)	HPLC-UV analysis	In 2 samples: 4.0 µg/l (light beer), 7.8 µg/l (dark beer)	Veršilovskis et al. (2008b)
Peanut seed	Fruit=20 Roasted=20 Roasted with salt=20	60	Cyclohexane for 10 h using Soxlet type extractor	TLC	Roasted seed: 3 samples 16.8 µg/kg, 12.2 µg/kg, 14.8 µg/kg Roasted with salt seed: in 1 sample 12.2 µg/kg	Youssef et al. (2008)
Saudi city of Jeddah markets	Coffee bean seed	30	Chloroform and purified by phenyl-bond solid phase	TLC	In 3 samples: 13 ng/g, 11 ng/g, 5 ng/g	Bokhari and Aly (2009)
Mills	Wheat bran	8 isolates from 5 samples	Chloroform:methanol 2:1 v/v	TLC	All 7 isolates	Tančinová and Labuda (2009)
Latvia and Belgium local supermarkets	Cheese	Latvia = 8 Belgium = 13	Acetonitrile–water (90 : 10, v/v) and n-hexane	LC-MS/MS system	Latvia ‒ in 4 samples 2 × 0.04, 0.07, 0.03 (µg/kg). Belgium ‒ 3 samples 1.23, 0.03, 0.52 (µg/kg).	Veršilovskis et al. (2009)
Pig farms 2006–2009	Several samples from different materials	Wheat ‒ 93 Corn – 111 Barley – 146 Soybean – 15 Sunflower cake – 53 Mixed feed for piglets of group 0–2 months/2–4 months, sows and fattening – 120/94	_____	ELISA method	2008: 8% of corn samples; 2006:7–17% of wheat, soybean, pea, and mixed feeds for piglets of age groups 0–2 and 2–4 months; in 2007, 9–22% of samples of wheat, corn, barley, pea, bran, sunflower cake, and mixed feeds for piglets of age group 0–2 months and sows; in 2009, 8–50% of samples of all feed types, except mixed feeds for piglets of age group 2–4 months and sows	Kovalenko et al. (2011)
Foods and feedstuffs from Burkina Faso	Maize = 26, feed = 4 and others = 30	Total 122	Acetonitrile/water/acetic acid (79:20:1, v/v/v)	LC-MS/MS analysis	Maize: 2 samples, median 2.3 µg/kg Feed: 3, median 6.5 µg/kg Others 2 median 6.7 µg/kg	Warth et al. (2012)
Foods and feedstuffs from Mozambique	Maize = 13, groundnuts = 23, feed = 10 and others = 7				Maize: 1, median 2.7 µg/kg. Groundnuts: 1, median 9.7 µg/kg. Feed: 1, median 11 µg/kg. Others: 2, median 26.1 µg/kg	
Bread samples from Riga local supermarkets	Plain rye bread	6	16% water in acetonitrile	LC-MS/MS and ESI	In 1 sample = 2.4 μg/kg	Veršilovskis et al. (2012)
	Mixed rye-wheat bread	9			In 1 sample = 7.1 μg/kg	
	Plain wheat bread	14			In 3 samples = 4.4 μg/kg; 3.2 μg/ kg; 5.6 μg/kg	
Different locations in Riyadh city, Kingdom of Saudi Arabia	Almond seed	20	Acetone, chloroform	HPLC	300–440 ppb	Yassin et al. (2013)
India rice	Rice	36	Chloroform/methanol (2:1, v/v), mycelia	TLC	5 isolates out of 12	Sawane A and Sawane M (2014)
Animal Feed	Straw	149	Acetonitrile/water (84/16) containing 1% formic acid	LC-MS/MS analysis	2 samples 0.001–0.038 mg/kg	Mol et al. (2014)
	Hay	43			22 samples 0.0012–0.17 mg/kg	
2 beef cattle farms	Grass	106 samples in 2011 and 69 samples in 2014	Acetonitrile/water/acetic acid (79:20:1, *v*/*v*/*v*).	LC-MS/MS System with ESI source HPLC System	90% in 2011 and 60% in 2014	Nichea et al. (2015)
143 rice processors	Milled rice	38	Acetonitrile/water/acetic acid (79:20:1,v/v/v)	LC-MS/MS with ESI source and HPLC System	50% of rice samples, mean value 19 μg/kg, maximum value 125 µg/kg	Rofiat et al. (2015)
Domestic and imported cereals and cereal products from different countries in different regions within the European Union	Cereal grains: Wheat Rye Maize Rice Barley Oats	221 35 33 28 59 51	Acetonitrile-water 80+20 v/v	LC-MS/MS analysis	Wheat: 12 samples (9 = LOD-0.5 µg/kg, 3 = 0.5–1.5 µg/kg); rye: 2 samples (1 = LOD-0.5 µg/kg, 1 = 0.5–1.5 µg/kg); maize: 2 samples (0.5–1.5 µg/kg); Rice: 27 samples (8 = LOD-0.5 µg/kg, 8 = 0.5–1.5 µg/kg, 10 = 1.5–5 µg/kg, 1 = 5 µg/kg); barley: 1 sample (1.5–5 µg/kg); oats: 11 samples (4 = LOD-0.5 µg/kg, 5 = 0.5–1.5 µg/kg, 1 = 1.5–5 µg/kg, 1 = 5 µg/kg)	Mo et al. (2015)
	Cereal products: Grain milling products, Rice, Pasta, Bread and rolls, Breakfast cereals, Fine bakery ware, Cereal-based infant food	125 89 115 143 97 90 54			Grain milling products: 6 samples (5 = LOD-0.5 µg/kg, 1 = 0.5–1.5 µg/kg); rice: 19 samples (12 = LOD-0.5 µg/kg, 5 = 0.5–1.5 µg/kg, 2 = 1.5–5 µg/kg); pasta: 6 samples LOD-0.5 µg/kg; bread/rolls: 10 samples (6 = LOD-0.5 µg/kg, 2 = 0.5–1.5 µg/kg, 2 = 1.5–5 µg/kg); breakfast cereals: 18 samples (11 = LOD-0.5 µg/kg, 6 = 0.5–1.5 µg/kg, 1 = 1.5–5 µg/kg); fine bakery ware: 6 samples (5 = LOD-0.5 µg/kg, 1 = 0.5–1.5 µg/kg); cereal-based infant food: 4 samples (2 = LOD-0.5 µg/kg, 2 = 0.5–1.5 µg/kg);	
					22 samples 0.0012–0.17 mg/kg	
Philippines coffee beans from production	Coffee beans	42	Chloroform	TLC and HPLC	Mean concentrations 55.8, 161.7 and 193.7 μg/kg	Culliao and Barcelo (2015)
Farms and storage facilities	Paddy rice	49	Acetonitrile-water 80/20v/v	LC-MS/MS system.	All samples in the range of 0.29–15.85 μg/kg	Bertuzzi et al. (2017)
Retail and wholesale resources	Processed rice	83			In 21% of samples (*n* = 19) 0.12 and 1.32 μg/kg (brown rice)	

Liquid chromatography-tandem mass spectrometry was used for STC detection in nine studies with limits of detection (LOD) ranging from 0.03 to 2.0 μg/kg and five out of nine had also limits of quantification (LOQ) ranging from 0.1 to 0.5 µg/kg (Warth et al. 2012; Veršilovskis et al. 2008a; b, 2009; Veršilovskis and Bartkevičs 2012; Mol et al. 2014; Rofiat et al. 2015; Nichea et al. 2015; Bertuzzi et al. 2017). Also thin-layer chromatography (El-Shanawany et al. 2005; Labuda and Tančinová 2006; Youssef et al. 2008; Bokhari and Aly 2009; Tančinová and Labuda 2009; Sawane and Sawane 2014; Culliao and Barcelo 2015), high-performance liquid chromatography (Veršilovskis et al. 2008a; b; Yassin et al. 2013; Culliao and Barcelo 2015) and enzyme-linked immunoassay (Kovalenko et al. 2011) analytical methods were used to determine STC in samples. However, in some of these studies, LOD or LOQ values were not available.

## 6. Climate change influence on STC production

Climate change has been occurring since the earth existed, and global temperatures normally show that 7 of the top 10 warmest years on record have occurred since the 1990s. The decade of 2000–2009 was the warmest period worldwide (EPA 2010). By 2100, the atmospheric concentration of CO_2_ is predicted to rise up to the range of 540 and 970 ppm above the current concentration. Together with other greenhouse gases such as CH_4_, this will lead to a predicted global temperature increase of 1.1‒6.4°C, depending on different models used and global region (In et al. 2007; Battilani et al. 2012). There is increased risk of European countries with temperate climates to have the higher exposure to fungi and mycotoxins due to climate change, which has already been identified by some authors (Paterson and Lima 2011; Battilani et al. 2012). The climate of these countries will probably become warmer reaching temperatures of 33°C, which is, for instance, a temperature very close to the optimal temperature for *Aspergillus* section *Versicolores* growth (30°C) (Atalla et al. 2003) and STC production (optimal temperature between 23°C and 29°C) (Atalla et al. 2003). However, effects of climate change on fungal species distribution and activity are difficult to predict because they are influenced in many different ways such as fungal characteristics, host features and availability, and competitive interactions between microbiota. In addition, environmental variables such as temperature, water availability and atmospheric CO_2_ and the interaction of these variables make it difficult to predict their influence on fungal distribution (Boddy 1984) and, consequently, mycotoxin presence in food and feed even higher (In et al. 2007). Mycotoxins are profoundly dependent on climate, plant and storage-associated problems, and also influenced by non-infectious factors (e.g. bioavailability of (micro)nutrients, insect damage, and other pests attack) that are also driven by climatic conditions. Therefore, climate represents the crucial factor for agro-ecosystem powering fungal colonisation and mycotoxin production (Magan et al. 2003).

## 7. Future perspectives regarding food and feed contamination

The report by the CONTAM Panel from EFSA mentioned the need for more occurrence data on STC in food and feed across European countries to allow an accurate assessment of dietary exposure. Furthermore, the prediction of climate change and how it can influence fungal contamination and mycotoxin production should be considered. Therefore, besides not knowing in detail what is the actual exposure to this mycotoxin in Europe, the new scenario of climate change brings new challenges due to a probable new exposure trends, particularly in countries with temperate climate.
